# CoXpress: differential co-expression in gene expression data

**DOI:** 10.1186/1471-2105-7-509

**Published:** 2006-11-20

**Authors:** Michael Watson

**Affiliations:** 1 Informatics Group, Institute for Animal Health, Compton, Newbury, Berks RG20 7NN, UK

## Abstract

**Background:**

Traditional methods of analysing gene expression data often include a statistical test to find differentially expressed genes, or use of a clustering algorithm to find groups of genes that behave similarly across a dataset. However, these methods may miss groups of genes which form differential co-expression patterns under different subsets of experimental conditions. Here we describe coXpress, an R package that allows researchers to identify groups of genes that are differentially co-expressed.

**Results:**

We have developed coXpress as a means of identifying groups of genes that are differentially co-expressed. The utility of coXpress is demonstrated using two publicly available microarray datasets. Our software identifies several groups of genes that are highly correlated under one set of biologically related experiments, but which show little or no correlation in a second set of experiments. The software uses a re-sampling method to calculate a p-value for each group, and provides several methods for the visualisation of differentially co-expressed genes.

**Conclusion:**

coXpress can be used to find groups of genes that display differential co-expression patterns in microarray datasets.

## Background

Microarrays have become a standard tool for the exploration of global gene expression changes at the cellular level [1]. Data analysis often includes the use of a statistical test, such as a t-test or analysis of variance, to find genes differentially expressed in one set of conditions when compared to another, or the use of clustering algorithms in order to find groups of genes which behave similarly over a number of experiments [2]. However, these techniques may not detect differential co-expression patterns that exist between two biological states.

Statistical tests, such as the t-test or ANOVA, identify genes that are differentially expressed under one or more conditions. The output of such tests is a simple list of genes, with an associated test statistic and p-value [3]. There is no indication of which genes may be interacting with one another. Alternatively, clustering algorithms are often used to find groups of genes which display similar expression profiles across a dataset, and these clusters are subsequently analyzed visually for patterns of interest [4,5]. Eisen *et al *used hierarchical cluster analysis to determine groups of co-expressed genes, and found that genes within those groups were functionally related [4], and the use of hierarchical cluster analysis is now a standard technique for analysing microarray data [2,6]. Yeung *et al *[7] assessed the use of hierarchical clustering to find groups of co-regulated genes. Various clustering algorithms were used on a number of datasets, and the results evaluated by determining those genes that share a common transcription factor. Of the algorithms tested, MCLUST [8] and two hierarchical methods (based on the pearson correlation coefficient) showed the highest coincidence of correlated and co-regulated genes.

However, genes which show highly correlated patterns of expression in one biological state, but not in another, may not be highly correlated across the entire dataset, and therefore would not be associated with one another if a clustering algorithm is used. Variation may exist in the expression of a gene in different groups of individuals due to the presence of sub-populations, and this may lead to that gene being grouped incorrectly. Furthermore, clustering algorithms do not provide methods to identify groups that are behaving differently in different biological conditions.

Recent work has concentrated on alternative approaches to the discovery of co-expressed genes. Li [9] describes a method whereby genes whose expression is associated with differential co-expression patterns in other pairs of genes may be discovered, and Lai *et al *[10] describe a conceptually similar method whereby pairs of genes that display differential co-expression patterns between the normal and cancerous state may be discovered. Other approaches have centred on the construction of large gene co-expression networks. Lee *et al *[11] analysed 60 human microarray data sets to construct gene co-expression networks conserved across multiple data sets, and Stuart *et al *[12] constructed a gene co-expression network across different organisms, indicating that such relationships are evolutionarily conserved. However, neither of the above attempted to find group of genes differentially co-expressed between different conditions. Choi *et al *[13] tackled this problem by constructing normal and tumour co-expression networks from a variety of public datasets, comparing the results to find differences in co-expression patterns associated with cancer. In all of these cases, the networks were built by comparing genes pairwise, using some variation of the pearson correlation coefficient, to determine if a co-expression link exists between the two genes. These links were then joined to form a co-expression network.

Cluster analysis and network construction can be thought of as alternative methods for finding co-expressed genes. Whereas networks concentrate on conserved, pairwise comparisons, there is no guarantee that genes that are close in the network, but are not directly linked, have correlated expression profiles. Alternatively, cluster analysis produces groups of genes that are correlated above a certain level, defined by where the tree is cut and the clustering algorithm, but there is no indication of which particular pairs of genes are interacting. Kostka and Spang [14] described the first method to investigate differentially co-expressed groups of genes, using an additive model for scoring gene-gene co-expression and then a stochastic search algorithm to find groups of genes showing differential co-expression patterns. Jen *et al *[15] have produced ACT, the Arabidopsis Co-expression Tool, which allows users to calculate co-expression across user-defined data sets and uses a correlation cut-off to define groups of genes.

Here we describe coXpress, a simple and easy to use package that allows users to explore differential co-expression in an intuitive way. The package is aimed at biologists who want to analyse differential co-expression in their data set, which can be achieved in just 4 simple commands once the data has been loaded. CoXpress uses hierarchical cluster analysis to explore the relationship between genes, cutting the tree to form groups of genes that are co-expressed. This is an intuitive approach that many biologists are familiar with. CoXpress then uses a resampling approach to find those groups that are co-expressed in one set of experiments and not in another. The package should be used as first step in the analysis of co-expression, and is designed to complement the approaches described above.

## Implementation

CoXpress is released as a package for R. R is a freely available, open-source statistical package [16] that is widely used in the biological community. R has very powerful statistical and graphical capabilities, and many add-on packages are freely available. The bioconductor project [17,18] provides a huge number of add-on packages for R, covering a wide range of biological data analysis applications, and the implementation of coXpress in R provides seamless integration with many of these packages. CoXpress is written in the native R language and has been fully tested on both windows and linux. R is available for windows, linux, unix and MacOS (including MacOS X).

The input for coXpress is a matrix of data, with rows representing genes and columns representing microarrays. The R data.frame object is most convenient, and can be created by reading in a text file (using the **read.table **function), an Excel spreadsheet (using the RODBC library) or from existing R objects, created by the packages from the bioconductor project such as affy [19], limma [20] or marray [18].

The genes are first clustered based on their expression values in a subset of experiments (termed subset 1), using the **cluster.gene **function. This function wraps the **dist**, **cor **and **hclust **functions that are built in to R, and thus provides a simple interface to hierarchical clustering. When a correlation coefficient is used as the distance measure, the distance measure is calculated as 1 - *r*, where *r *is the pearson correlation coefficient. The resulting tree is cut at a user-defined value, using the **cutree **function, to form groups of genes that are co-expressed in subset 1. These groups are then examined in both subset 1 and a second set of experiments, defined by the user, which we will term subset 2.

Groups of size 1 are ignored as there can be no co-expression. Groups of size two are handled by the **cox.pairs **function. The **cox.pairs **function uses the **cor.test **function in R to test if the genes are significantly correlated in subset 1 and subset 2. Thus, a pair of genes significantly correlated in subset 1 and not significantly correlated in subset 2 can be described as differentially co-expressed.

**Figure 1:**
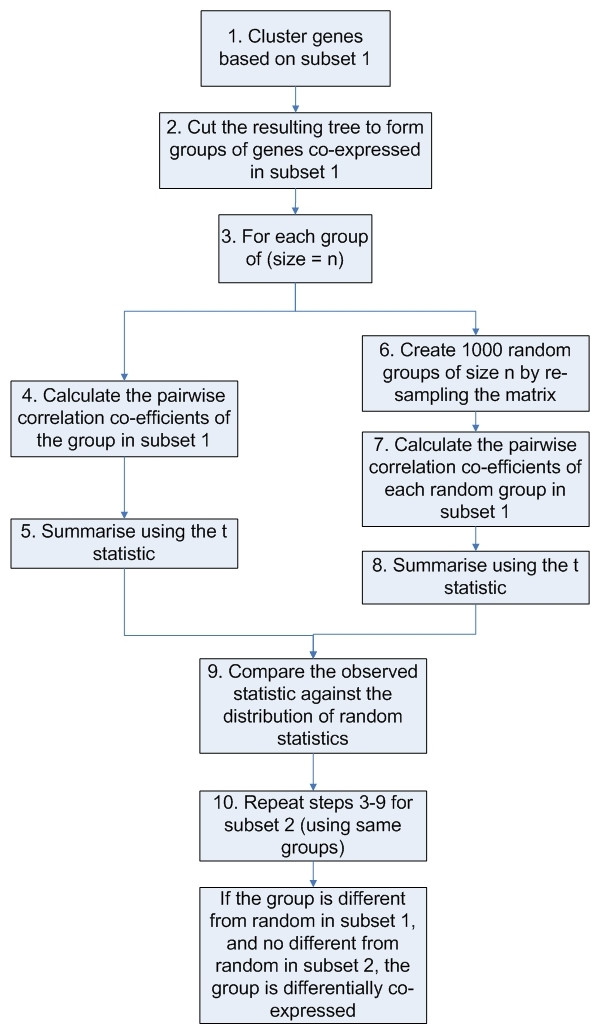
**Conceptual flow of analysis through the coXpress function**. This figure shows the conceptual steps taken by the coXpress function in order to find differentially co-expressed groups of genes in microarray data. Steps should be followed in numerical order.

Groups with more than two members are handled by the **coXpress **function. The flow of analysis in **coXpress **is represented in figure 1. For each group of size *n*, where *n *≥ 3, the pairwise correlation coefficients of the group in subset 1 are calculated. These are then summarised using the t-statistic, the use of which is discussed below. Then, *m *random groups of size *n *are created by randomly re-sampling the data matrix. For each of these random groups, the pairwise correlation coefficients of the group in subset 1 are calculated and again summarised using the t statistic. Thus, a distribution of t statistics is created, of size *m*, from randomly assigned groups of size *n*. The observed t statistic is then compared to the distribution of random t statistics. The proportion of random statistics greater than the observed is used as a "probability of randomness" for the group in subset 1. This process is then repeated for subset 2. A group which is found to be non-random in group 1 and random in group 2 is said to be differentially co-expressed. These groups will be highly correlated in subset 1 but show little or no correlation in subset 2. To find the reverse, the process must be repeated, but basing the original groups on a cluster analysis of the data based on subset 2.

The t-statistic is used here not as a test of significance, but as a means of summarising a set of pairwise correlation coefficients into a single value. Correlation coefficients are on the scale:

-1 ≤ *r *≤ +1

where 1 represents positive correlation, -1 represents negative correlation and 0 represents no relationship. The t-statistic is used here to summarise the "difference from zero" of a group of pairwise correlation coefficients. The exact formula for this is:

t=x¯se(x)
 MathType@MTEF@5@5@+=feaafiart1ev1aaatCvAUfKttLearuWrP9MDH5MBPbIqV92AaeXatLxBI9gBaebbnrfifHhDYfgasaacH8akY=wiFfYdH8Gipec8Eeeu0xXdbba9frFj0=OqFfea0dXdd9vqai=hGuQ8kuc9pgc9s8qqaq=dirpe0xb9q8qiLsFr0=vr0=vr0dc8meaabaqaciaacaGaaeqabaqabeGadaaakeaacqWG0baDcqGH9aqpdaWcaaqaaiqbdIha4zaaraaabaGaem4CamNaemyzauMaeiikaGIaemiEaGNaeiykaKcaaaaa@36B1@

where *x *is the vector of unique, pairwise correlation coefficients, x¯
 MathType@MTEF@5@5@+=feaafiart1ev1aaatCvAUfKttLearuWrP9MDH5MBPbIqV92AaeXatLxBI9gBaebbnrfifHhDYfgasaacH8akY=wiFfYdH8Gipec8Eeeu0xXdbba9frFj0=OqFfea0dXdd9vqai=hGuQ8kuc9pgc9s8qqaq=dirpe0xb9q8qiLsFr0=vr0=vr0dc8meaabaqaciaacaGaaeqabaqabeGadaaakeaacuWG4baEgaqeaaaa@2E3D@ is the mean of x and *se*(*x*) denotes the standard error of *x*. A group of highly correlated genes will have a mean correlation close to 1 and a small standard error, resulting in a large value for *t*. However, a group of uncorrelated genes will have a mean close to 0 and a relatively large standard error, resulting in a small value for *t*. The observed t statistic is compared against *m *random t statistics in order to calculate a probability of randomness.

## Results

### The AML/ALL leukaemia dataset

The utility of coXpress is demonstrated using gene expression data from the leukaemia microarray study of Golub *et al *[21]. This dataset represents gene expression measurements from 38 tumour mRNA samples, 27 acute lymphoblastic leukaemia (ALL) cases and 11 acute myeloid leukaemia (AML) cases. The HU6800 Affymetrix array was used, which contains 6800 probesets. The dataset has been filtered such that genes with negative values in any sample have been removed, resulting in 2568 genes.

Using coXpress, the genes were first clustered according to their expression levels in the 27 ALL samples, using the **cluster.gene **function. The distance measure used was *1 - r*, where *r *is the pearson correlation coefficient. The resulting tree was cut at a distance of 0.4, representing a correlation coefficient of 0.6, using the **cutree **function.

These groups were then examined in both the ALL and AML cases using the **coXpress **function. The observed t statistics in all cases were compared with the t statistics generated by randomly resampling the dataset 10,000 times for each group size. The resulting table contains one row for each group.

To test the robustness of the method to outliers, a bootstrapping approach was used. Each group was re-tested 1000 times, each time randomly selecting 75% of the observations for each leukaemia subtype (20 AML cases and 8 AML cases). The number of times each group was found to be differentially co-expressed by the coXpress method was recorded.

Table 1 shows the results filtered for groups that are non-random in the ALL subset, random in the AML subset, and with more than 6 members. As can be seen, there are 10 groups, varying in size from 7 to 34 members. The mean pairwise correlations for the groups are all above 0.6 in the ALL cases, yet show little or no correlation in the AML cases, with mean values ranging from -0.093 to 0.144. The robustness resampling method provides evidence that the groups found are robust to outliers, with nine out of ten groups being found in over 90% of the resampled data sets, and the other being found in 76%.

**Table 1:** Differentially co-expressed groups from the Golub dataset

group	N	t1	t2	pr.g1	pr.g2	mean.corr1	mean.corr2	robustness
3	34	155.6977	0.175907	0	0.4439	0.70126	0.002587	901
62	7	59.75635	-1.06598	0	0.876	0.7201	-0.09352	1000
121	11	54.77229	1.835753	0	0.065	0.679399	0.085609	926
21	12	51.18428	-0.83765	0	0.8032	0.659053	-0.03528	1000
79	7	50.37868	0.830252	0	0.2021	0.724548	0.063511	914
78	10	50.376	1.040897	0	0.1614	0.655953	0.049546	950
131	8	42.89021	1.744949	0	0.0787	0.668891	0.144298	760
157	7	41.89854	-0.1536	0	0.5177	0.696409	-0.01012	1000
472	7	36.4184	-0.56951	0	0.6958	0.642614	-0.04193	1000
193	7	32.0097	-0.06639	0	0.4814	0.707166	-0.00404	974

Differentially co-expressed groups from the ALL/AML dataset of Golub *et al *[21]. Group is the group number, N is the group size, t1 and t2 are the observed t-statistics in the ALL and AML subsets, pr.g1 and pr.g2 are the probability of randomness statistics for the ALL and AML subsets, mean.corr1 and mean.corr2 are the mean pairwise correlation coefficients for the genes in the ALL and AML cases and robustness is the number of times that the group was differentially co-expressed in 1000 randomly resampled data sets using only 75% of the observations in each leukaemia subtype. Groups are ordered by t1. The table has been filtered such that pr.g1 ≤ 0.05, pr.g2 ≥ 0.05 and N ≥ 7.

**Figure 2:**
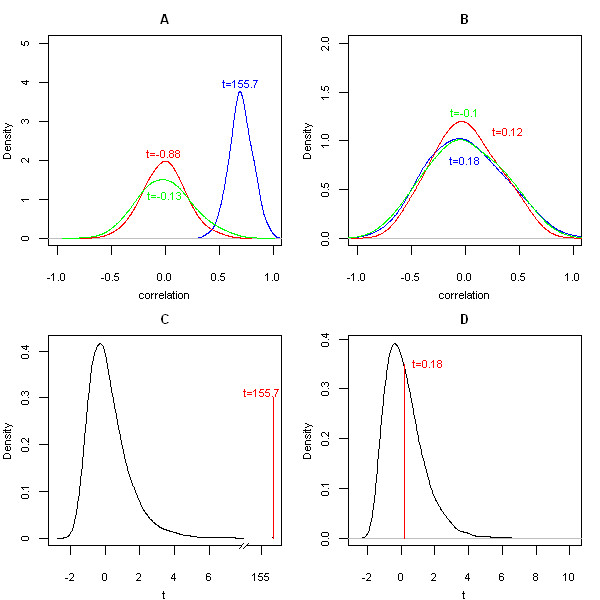
**A differentially co-expressed group from the Golub dataset compared to random distributions**. Group 3 (n = 34) from the Golub [21] data compared to random distributions. A) the distribution of pairwise correlation coefficients for group 3 in the ALL subset (blue) compared to the distribution of pairwise correlation coefficients from a group of the same size generated by the random uniform distribution (red) and the distribution of pairwise correlation coefficients from a group of the same size randomly selected from the dataset (green). B) Equivalent graph to A for the AML subset. C) A comparison of the observed t-statistic for group 3 in the ALL subset with a distribution of 10,000 random t-statistics generated by randomly resampling the dataset. D) Equivalent graph to C for the AML subset. Distributions were smoothed and drawn using density function in R [16]. Note that graph C has a broken x-axis.

Figure 2 demonstrates the method of coXpress. These graphs show data from the largest of the groups, group 3, which has 34 members. Fig. 2A compares the distribution of pairwise correlation coefficients in the ALL subset with two random distributions. The blue graph is the distribution of observed correlation coefficients in the ALL subset for group 3, the red graph is the distribution of pairwise correlation coefficients from data generated by the random uniform distribution, and the green graph is the distribution of pairwise correlation coefficients from a group of genes randomly selected from the dataset. As can be seen, the observed distribution for this group in the ALL subset is very different from the two random distributions. Fig. 2B is an identical graph for the group based on the AML subset. This time, the observed distribution shows no difference compared to the two random distributions. The t-statistics for each distribution are shown on these graphs. Fig. 2C shows the observed t-statistic for group 3 in the ALL subset compared to the distribution of 10,000 randomly generated t-statistics, and Fig. 2D is the equivalent graph for the AML subset. Again, it is clear that this group in the ALL subset is non-random, yet is no different to random in the AML subset.

**Figure 3:**
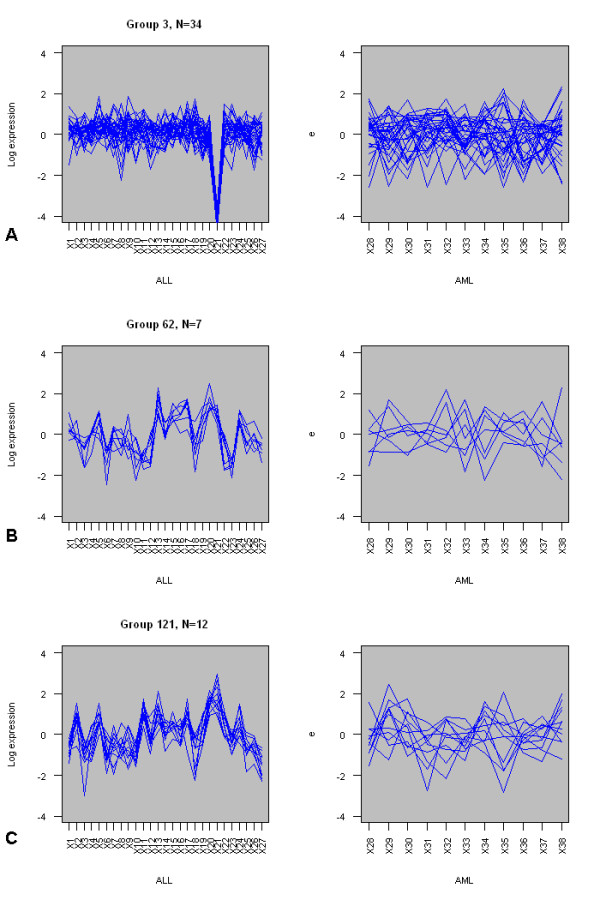
**Expression profiles for three differentially co-expressed groups in the Golub dataset**. Expression profiles for three groups of differentially co-expressed genes from the Golub dataset [21]. A) Group 3 (n = 34) in 27 ALL samples (left) and 11 AML samples (right). B) Group 62 (n = 7) in 27 ALL samples (left) and 11 AML samples (right). C) Group 121 (n = 12) in 27 ALL samples (left) and 11 AML samples (right). Expression levels have been scaled and centred.

Figure 3 shows the top 3 groups in table 1 graphically. Fig. 3A is the largest of the groups, with 34 members. These 34 genes have a mean pairwise correlation of 0.70 in the ALL subset, but only 0.003 in the AML subset. Fig. 3B shows a smaller group, with 7 members, with a mean pairwise correlation of 0.72 in the ALL subset and -0.09 in the AML subset. Finally, fig. 3C shows a group with 11 members, with a mean pairwise correlation coefficient of 0.679 in the ALL subset and only 0.086 in the AML subset. These graphs were produced using the **plot.compare.group **and **plot.cluster.genes **functions.

**Figure 4:**
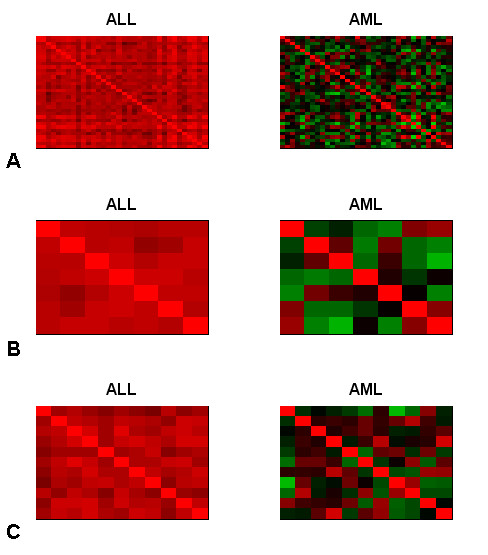
**Images of the correlation matrices for three differentially co-expressed groups in the Golub dataset**. Images of the correlation matrices for three groups of differentially co-expressed genes from the Golub dataset [21]. A) Group 3 (n = 34) in 27 ALL samples (left) and 11 AML samples (right). B) Group 62 (n = 7) in 27 ALL samples (left) and 11 AML samples (right). C) Group 121 (n = 12) in 27 ALL samples (left) and 11 AML samples (right). Each coefficient in the correlation matrix is represented as a square, with the colour of the square representing the amount of correlation. The colour scale used is green to red, with green representing -1, red representing +1 and black representing 0.

Figure 4 shows the same three groups in a different way. Here, each plot is a representation of the correlation matrix of the group of genes in either the ALL or the AML subsets. Each coefficient in the correlation matrix is represented as a square, with the colour of the square representing the amount of correlation. The colour scale used is green to red, with green representing -1 (negative correlation), red representing +1 (positive correlation) and black representing 0 (no correlation). In all three groups, the correlation matrices are red for the ALL subset, yet are a mixture of black, green and red in the AML subset. This view of the data is more useful than simply considering the average pairwise correlation, as it shows all of the values in an intuitive way. These graphs were produced using the **show.cor.matrices **function.

In each of the differentially co-expressed groups, not all pairwise correlation coefficients will have decreased or changed. To examine which pairs of genes have changed, the **inspect.group **function should be used. Table 2 shows the ten pairwise correlation coefficients that have changed the most between the ALL and AML subsets in group 62. As can be seen, these pairs of genes are all positively correlated in the ALL subset but are negatively correlated in the AML subset. Table 3 shows the ten pairwise correlation coefficients that have changed the least between the ALL and AML subsets in group 62. Many of these pairs of genes are still positively correlated in the AML subset, but not to the same extent. It is important that each differentially co-expressed group is examined in this way to determine which of the pairs of correlated genes have changed and which have not.

**Table 2:** Most changed pairwise correlation coefficients between the ALL and AML subsets in group 62

GeneA	GeneB	Cor Group 1	Cor Group 2
X82200_at	U72936_s_at	0.75613	-0.68659
D87127_at	U72936_s_at	0.772053	-0.49596
HG1400-HT1400_s_at	U72936_s_at	0.741713	-0.50531
D87127_at	Y08614_at	0.741222	-0.47561
X82200_at	Y08614_at	0.783967	-0.40329
X82200_at	Z26491_s_at	0.773757	-0.39009
D14043_at	HG1400-HT1400_s_at	0.654386	-0.48988
D14043_at	Y08614_at	0.687928	-0.39583
D87127_at	Z26491_s_at	0.624918	-0.39106
D14043_at	D87127_at	0.739456	-0.24224

The ten pairwise correlation coefficients that have changed the most between the ALL and AML subsets in group 62 of the Golub [21] data set. GeneA and GeneB refer to the names of the genes, Cor Group 1 refers to their correlation coefficient in the ALL subset and Cor Group 2 refers to their correlation coefficient in the AML subset.

**Table 3:** Least changed pairwise correlation coefficients between the ALL and AML subsets in group 62

GeneA	GeneB	Cor Group 1	Cor Group 2
D14043_at	X82200_at	0.710406	-0.11507
HG1400-HT1400_s_at	Z26491_s_at	0.730394	0.014146
Y08614_at	HG1400-HT1400_s_at	0.802284	0.131596
Y08614_at	U72936_s_at	0.720434	0.05055
X82200_at	HG1400-HT1400_s_at	0.687068	0.222248
D87127_at	X82200_at	0.727349	0.375199
D14043_at	Z26491_s_at	0.718968	0.484247
Z26491_s_at	U72936_s_at	0.679597	0.511949
D87127_at	HG1400-HT1400_s_at	0.577312	0.440534
D14043_at	U72936_s_at	0.708798	0.586629

The ten pairwise correlation coefficients that have changed the least between the ALL and AML subsets in group 62 of the Golub [21] data set. GeneA and GeneB refer to the names of the genes, Cor Group 1 refers to their correlation coefficient in the ALL subset and Cor Group 2 refers to their correlation coefficient in the AML subset.

The **GOHyperG **function of the **GOstats **package [22] was used to find GO terms over-represented in the differentially co-expressed groups. Group 3, with 34 members, is enriched for GO terms for lymph node development, cell organisation and biogenesis, and protein biosynthesis and transport. Group 62, which has 7 members, is enriched for GO terms for methyltransferase activity, DNA modification, protein transport and DNA and protein methylation. Group 121, with 11 members, is enriched for GO terms for nucleotidase activity, and RNA splicing, processing and metabolism.

### The ALL subtype dataset

This dataset is from the Acute Lymphoblastic Leukaemia study by Yeoh *et al *[23]. Six subtypes of ALL leukaemias are represented in 248 cases. The six subtypes are T-ALL, E2A-PBX1, BCR-ABL, TEL-AML1, MLL rearrangement, and hyperdiploid >50. The HG_U95Av2 Affymetrix microarray was used which contains 12,600 probesets. The dataset has been filtered such that genes with negative values in any sample have been removed, resulting in 1516 genes present in the dataset.

Using coXpress, the genes were first clustered according to their expression levels in the BCR-ABL samples, using the **cluster.gene **function. The distance measure used was *1 - r*, where *r *is the pearson correlation coefficient. The resulting tree was cut at a distance of 0.5, representing a correlation coefficient of 0.5, using the **cutree **function. These groups were then examined in both the BCR-ABL and T-ALL subsets.

Those groups of size two were analysed using the **cox.pairs **function. Table 4 shows three pairs of genes that are significantly positively correlated in the BCR-ABL subset, and significantly negatively correlated in the T-ALL subset.

**Table 4:** Differentially co-expressed pairs in the ALL subtype dataset

group	N	r1	p1	r2	p2
14	2	0.658202	0.007638	-0.58144	4.33E-05
201	2	0.655991	0.007916	-0.44687	0.002664
143	2	0.67791	0.00548	-0.30901	0.043776

Differentially co-expressed pairs of genes from the ALL subtype dataset [23]. Group is the group number, N is the group size, r1 and r2 are the observed pearson correlation coefficients in the BCR-ABL and T-ALL1 subsets, and p1 and p2 are the corresponding p-values.

Groups of N ≥ 3 were analysed in the BCR-ABL and T-ALL subsets using the **coXpress **function. The observed t statistics in all cases were compared with the t statistics generated by randomly resampling the dataset 10,000 times for each group size. The resulting table contains one row for each group.

To test the robustness of the method to outliers, a bootstrapping approach was used. Each group was re-tested 1000 times, each time randomly selecting 75% of the observations for each leukaemia subtype. The number of times each group was found to be differentially co-expressed by the coXpress method was recorded.

**Figure 5:**
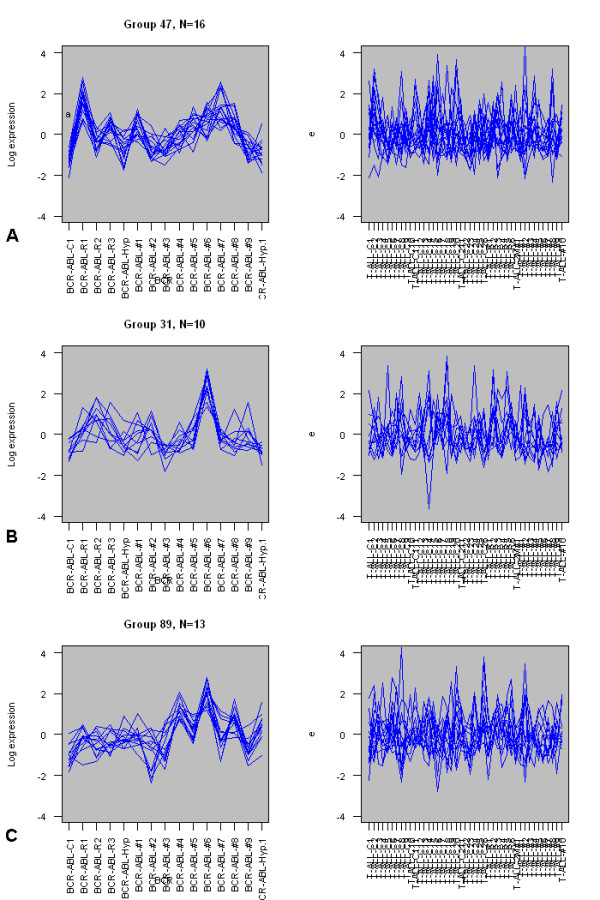
**Expression profiles for three differentially co-expressed groups in the ALL subtype dataset**. Expression profiles for three groups of differentially co-expressed genes from the Yeoh *et al *dataset [23]. A) Group 47 (n = 16) in 15 BCR-ABL samples (left) and 43 T-ALL1 samples (right). B) Group 31 (n = 10) in 15 BCR-ABL samples (left) and 43 T-ALL1 samples (right). C) Group 89 (n = 13) in 15 BCR-ABL samples (left) and 43 T-ALL1 samples (right). Expression levels have been scaled and centred.

Table 5 shows the results filtered for groups that are non-random in the BCR-ABL cases, random in the T-ALL cases, and with more than 10 members. Figure 5 shows the top 3 groups in table 1 graphically. Figure 5A shows a group of 16 genes that have a mean pairwise correlation coefficient of 0.669 in the BCR-ABL subset, yet only 0.06 in the T-ALL subset. Figure 5B shows a group of 10 genes that have a mean correlation of 0.65 in the BCR-ABL subset and only 0.08 in the T-ALL data. Finally, Figure 5C shows a group of 13 genes that have an average correlation of 0.64 in the BCR-ABL data, yet only 0.04 in the T-ALL data. The robustness resampling method provides evidence that the groups found are robust to outliers, with twelve out of thirteen groups being found in over 80% of the resampled data sets, and the other being found in 68.6%.

**Table 5:** Differentially co-expressed groups from the ALL subtype dataset

group	N	t1	t2	pr.g1	pr.g2	mean.corr1	mean.corr2	robustness
47	16	59.8611	2.453073	0	0.0684	0.66915	0.05863	805
31	10	29.20261	2.194293	0	0.0677	0.64726	0.07787	861
89	13	42.1617	1.934779	0	0.0904	0.63834	0.04420	885
41	11	30.19613	1.122452	0	0.2115	0.63042	0.05146	986
25	12	34.18317	2.531833	0	0.0503	0.62832	0.10027	686
9	14	30.35473	2.324388	0	0.0655	0.59705	0.06109	829
71	13	33.96871	2.236447	0	0.0658	0.58783	0.05880	855
15	19	45.6475	1.865767	0	0.1373	0.58500	0.03853	1000
103	10	29.54434	-1.24949	0	0.9613	0.58393	-0.03758	1000
19	20	45.45846	1.509674	0	0.2093	0.5810	0.02446	1000
11	16	43.62891	1.402566	0	0.1964	0.57995	0.02908	1000
114	12	32.84917	1.192507	0	0.205	0.57624	0.03668	995
32	13	34.2227	1.097651	0	0.2275	0.57004	0.03208	982

Differentially co-expressed groups from the ALL subtype dataset of Yeoh *et al *[23]. Group is the group number, N is the group size, t1 and t2 are the observed t-statistics in the BCR-ABL and T-ALL1 subsets, pr.g1 and pr.g2 are the probability of randomness statistics for the BCR-ABL and T-ALL1 subsets, mean.corr1 and mean.corr2 are the mean pairwise correlation coefficients for the genes in the BCR-ABL and T-ALL1 subsets and robustness is the number of times that the group was differentially co-expressed in 1000 randomly resampled data sets using only 75% of the observations in each leukaemia subtype. Groups are ordered by mean.corr1. The table has been filtered such that pr.g1 ≤ 0.05, pr.g2 ≥ 0.05 and N ≥ 10.

The **GOHyperG **function of the **GOstats **package [22] was used to find GO terms over-represented in the differentially co-expressed groups. Group 47 with 16 members, is enriched for GO terms for hormone catabolism, glucocorticoid receptor signalling and glucocorticoid catabolism. Group 31 with 10 members contains two probes for a gene in the RAS oncogene family, and is enriched for GO terms for oxidoreductase activity and ubiquitin activating enzyme activity. Finally, group 89 with 13 members contains genes annotated as B-cell lymphoma and cancer susceptibility genes, as well as genes enriched for GO terms for endothelial cell migration, regulation of cell motility and migration, angiostatin binding and regulation of blood vessel endothelial cell migration.

## Discussion

It is clear that coXpress is capable of finding differentially co-expressed groups of genes in both data sets. The groups presented above are extremely highly correlated in one subset of experiments, yet show little correlation in another subset. Furthermore, these patterns of correlation are shown to be non-random in the first subset, and no different from random in the second subset. The results show that it is the overall correlation structures of these groups that have changed significantly and some pairs of genes are still highly correlated in the second subset. It is important that each group is examined using the **inspect.groups **function in order to determine which of the pairs of genes are still correlated and which are not. The groups found by coXpress could also feed into the network construction technique described by Choi *et al *[13] to determine which pairwise relationships are conserved and which are not. One would expect the differences between ALL and AML leukaemia in the Golub dataset to be larger than those between different ALL subtypes in the Yeoh dataset, and the fact that coXpress can still find groups with such different correlation structures demonstrates the power of the method.

The use of hierarchical cluster analysis, followed by cutting the tree, is an intuitive approach and one that is familiar to biologists. However, it has limitations. For example, each gene may only be in one group, which does not ring true for biological systems, where many genes have multiple functions. Also, the choice of where to cut the tree is arbitrary. A high cut-off will produce many small groups of genes that are very highly correlated, whereas a lower cut-off will produce fewer groups, of larger size, which are not as highly correlated. In reality the user must use a range of different cut-offs to see which performs best with their dataset. Other clustering algorithms, such as MCLUST [7,8], have been shown to out-perform hierarchical cluster analysis, however, there is no reason why these algorithms could not be used to define the groups of genes prior to running the **coXpress **function.

There are several directions in which the software can be developed. At present, the user defines which subsets of experiments are analysed, however it is possible that coXpress could suggest, or improve, these groupings using an approach such as random forests or genetic algorithms. This may allow researchers to discover sub-populations in the system under study. Integration of other clustering algorithms with coXpress, such as MCLUST, may also improve the performance of the software. In particular, clustering or grouping algorithms that allow genes to be present in more than one group may be advantageous. Finally, the integration of network construction algorithms would allow researchers to further analyse and visualise the differentially co-expressed groups discovered by coXpress.

## Conclusion

We describe coXpress, an open-source R package that allows researchers to analyse differential co-expression patterns in DNA microarray data. CoXpress contains several methods for the discovery and visualisation of differentially co-expressed genes. We show how coXpress can be used to find groups of differentially co-expressed genes in two publicly available microarray datasets. The groups found are shown to be highly correlated in one subset of experiments, yet show little or no correlation in a second subset of experiments. A comparison against random distributions is used to obtain a p-value for the co-expression of the genes in different subsets.

## Availability and requirements

• **Project Name: **coXpress

• **Project Home Page: **

• **Operating Systems: **Windows, Linux

• **Programming Language: **R

• **Other Requirements: **R, gplots, gtools, gdata (for heatmaps), hu6800, hgu95av2, plotrix (for examples)

• **License: **GNU GPL

## Abbreviations

ALL: acute lymphoblastic leukaemia

AML: acute myeloid leukemia

T-ALL: T lineage leukaemias

E2A-PBX1: B lineage leukemias that contain t(1;19)

BCR-ABL: B lineage leukemias that contain t(9;22)

TEL-AML1: B lineage leukemias that contain t(12;21)

MLL rearrangement: B lineage leukemias that contain rearrangements in the *MLL *gene on chromosome 11, band q23

Hyperdiploid >50: hyperdiploid karyotype (i.e., >50 chromosomes)

## Authors' contributions

MW developed and tested the software in full.

## References

[B1] Schena M, Shalon D, Davis RW, Brown PO (1995). Quantitative monitoring of gene expression patterns with a complementary DNA microarray. Science.

[B2] Slonim D (2002). From patterns to pathways: gene expression data analysis comes of age. Nat Genet.

[B3] Stekel D (2003). Microarray Bioinformatics.

[B4] Eisen MB, Spellman PT, Brown PO, Botstein D (1998). Cluster analysis and display of genome-wide expression patterns. Proc Natl Acad Sci USA.

[B5] Spellman PT, Sherlock G, Zhang MQ, Iyer VR, Anders K, Eisen MB, Brown PO, Botstein D, Futcher B (1998). Comprehensive identification of cell cycle-regulated genes of the yeast Saccharomyces cerevisiae by microarray hybridization. Mol Biol Cell.

[B6] Quackenbush J (2001). Computational analysis of microarray data. Nat Rev Genet.

[B7] Yeung KY, Medvedovic M, Bumgarner RE (2004). From co-expression to co-regulation: how many microarray experiments do we need?. Genome Biol.

[B8] Yeung KY, Fraley C, Murua A, Raftery AE, Ruzzo WL (2001). Model-based clustering and data transformations for gene expression data. Bioinformatics.

[B9] Li KC (2002). Genome-wide coexpression dynamics: Theory and application. Proc Nat Acad Sci USA.

[B10] Lai Y, Wu B, Chen L, Zhao H (2004). A statistical method for identifying differential gene-gene co-expression patterns. Bioinformatics.

[B11] Lee HK, Hsu AK, Sajdak J, Qin J, Pavlidis P (2004). Coexpression analysis of human genes across many microarray data sets. Genome Res.

[B12] Stuart JM, Segal E, Koller D, Kim SK (2003). A gene-coexpression network for global discovery of conserved genetic modules. Science.

[B13] Choi JK, Yu U, Yoo OJ, Kim S (2005). Differential coexpression analysis using microarray data and its application to human cancer. Bioinformatics.

[B14] Kostka D, Spang R (2004). Finding disease specific alterations in the co-expression of genes. Bioinformatics.

[B15] Jen CH, Manfield IW, Michalopoulos I, Pinney JW, Willats WGT, Gilmartin PM, Westhead DR (2006). The Arabidopsis co-expression tool (ACT): a WWW-based tool and database for microarray-based gene expression analysis. The Plant Journal.

[B16] R. http://www.r-project.org.

[B17] Gentleman RC, Carey VJ, Bates DM, Bolstad B, Dettling M, Dudoit S, Ellis B, Gautier L, Ge Y, Gentry J, Hornik K, Hothorn T, Huber W, Iacus S, Irizarry R, Leisch F, Li C, Maechler M, Rossini AJ, Sawitzki G, Smith C, Smyth G, Tierney L, Yang JY, Zhang J (2004). Bioconductor: open software development for computational biology and bioinformatics. Genome Biol.

[B18] Bioconductor. http://www.bioconductor.org.

[B19] Gautier L, Cope L, Bolstad BM, Irizarry RA (2004). affy: analysis of Affymetrix GeneChip data at the probe level. Bioinformatics.

[B20] Smyth GK (2004). Linear models and empirical bayes methods for assessing differential expression in microarray experiments. Stat Appl Genet Mol Biol.

[B21] Golub TR, Slonim DK, Tamayo P, Huard C, Gaasenbeek M, Mesirov JP, Coller H, Loh ML, Downing JR, Caligiuri MA, Bloomfield CD, Lander ES (1999). Molecular classification of cancer: class discovery and class prediction by gene expression monitoring. Science.

[B22] Gentleman R (2004). Using GO for statistical analyses. Compstat 2004 Proceedings in Computational Statistics.

[B23] Yeoh EJ, Ross ME, Shurtleff SA, Williams WK, Patel D, Mahfouz R, Behm FG, Raimondi SC, Relling MV, Patel A, Cheng C, Campana D, Wilkins D, Zhou X, Li J, Liu H, Pui CH, Evans WE, Naeve C, Wong L, Downing JR (2002). Classification, subtype discovery, and prediction of outcome in pediatric acute lymphoblastic leukemia by gene expression profiling. Cancer Cell.

